# Developing a label propagation approach for cancer subtype classification problem

**DOI:** 10.3906/biy-2108-83

**Published:** 2021-12-20

**Authors:** Pınar GÜNER, Burcu BAKIR-GUNGOR, Mustafa COŞKUN

**Affiliations:** Department of Computer Engineering, Faculty of Engineering, Abdullah Gül University, Kayseri, Turkey

**Keywords:** Cancer subtype, bioinformatics, machine learning, label propagation, personalized medicine

## Abstract

Cancer is a disease in which abnormal cells grow uncontrollably and invade other tissues. Several types of cancer have various subtypes with different clinical and biological implications. Based on these differences, treatment methods need to be customized. The identification of distinct cancer subtypes is an important problem in bioinformatics, since it can guide future precision medicine applications. In order to design targeted treatments, bioinformatics methods attempt to discover common molecular pathology of different cancer subtypes. Along this line, several computational methods have been proposed to discover cancer subtypes or to stratify cancer into informative subtypes. However, existing works do not consider the sparseness of data (genes having low degrees) and result in an ill-conditioned solution. To address this shortcoming, in this paper, we propose an alternative unsupervised method to stratify cancer patients into subtypes using applied numerical algebra techniques. More specifically, we applied a label propagation-based approach to stratify somatic mutation profiles of colon, head and neck, uterine, bladder, and breast tumors. We evaluated the performance of our method by comparing it to the baseline methods. Extensive experiments demonstrate that our approach highly renders tumor classification tasks by largely outperforming the state-of-the-art unsupervised and supervised approaches.

## 1. Introduction

Cancer is a complex disease in which cells divide indefinitely and spread to the neighboring tissues. According to a report published by the World Health Organization (WHO) in 2018, around the world, 9.6 million people died due to cancer, and 18.1 million new cases were predicted **(**World Health Organization, 2018). There are several factors that cause cancer to be deadly. Tumor type, stage of cancer, clinical factors can be listed among many others. In order to improve the survival rates, cancer patients should be treated with the best possible plan based on the aforementioned factors. The area of precision medicine has been established to deal with personalized treatment with one of the objectives, personalized cancer treatment^1^. The first step of precision medicine applications is to stratify (cluster or categorize) cancer patients into meaningful subtypes based on the tumor molecular profiles and somatic mutations. Many cancer types have various subtypes where each of which has its own clinical implications such as patient survival time, drug response, and drug resistance. Thus, clear identification of cancer subtypes is crucial to determine the treatment steps, prognosis, and response to treatment (Hofree et al., 2013). As a result, separating patients into different groups based on their cancer subtypes can guide the selection of drugs that provide more effective outcomes and minimize the side effects. Since in vitro experiments and clinical trials are costly and time-consuming in order to facilitate the precision medicine applications, the number of computational studies, which tackle the cancer subtype identification problem has been soaring (Hofree et al., 2013; Jin et al., 2015; Kim et al., 2016; Cho et al., 2016; Zhang et al., 2018). Along this line, here we aim to develop an effective computational method for cancer subtype stratification. More specifically, we propose an alternative unsupervised computational method based on the idea of the sparsity of the given cancer data and by capitalizing applied numerical algebra techniques. We attempt to cluster tumors into meaningful subtypes with a better clustering accuracy as compared with the existing supervised and unsupervised approaches (Hofree et al., 2013; Zhang et al., 2018). To evaluate the proposed method, we used various cancer datasets, including colon, head and neck, uterine, bladder, and breast tumors, downloaded from the TCGA (The Cancer Genome Atlas) project (The Cancer Genome Atlas Research Network, 2013). Extensive experiments on these cancer datasets from TCGA show that our unsupervised stratification method significantly outperforms the state-of-the-art unsupervised methods (Hofree et al., 2013) for identifying cancer subtypes even its performance exceeds the supervised approaches (Zhang et al., 2018).

### 1.1. Related works

Large-scale cancer -omics studies aim to understand the molecular mechanisms of cancer, and they have demonstrated that cancer subtypes have a strong association with clinical outcomes. Several studies have been proposed to categorize the tumors into subtypes. In literature, cancer-subtyping studies can be basically divided into two categories: unsupervised and supervised techniques. In the context of subtype clustering, the difference between the supervised and stratification is mainly based on the usage of the subtype labels while the underlying algorithms perform smoothing of the somatic mutation data with gene-gene network. In other words, the smoothing is a simulation of a random walk on the gene-gene network from mutated genes, i.e, gene similarity (sharing common neighbors) in the graph is used to gain strength of similar somatic mutation profiles. If the method is using label information by forcing the random walker to walk towards the known label (by putting known subtype into the cluster centroid), these types of smoothing algorithms are categorized as supervised; otherwise, they are known as stratification. That is to say that the supervised classification and clustering algorithms show differences in terms of how they set the random walker to walk.

Moreover, different types of molecular data can be utilized for cancer subtype identification problem. Some of these studies stratify tumors using molecular profiles obtained from mRNA expression (Konstantinopoulos et al., 2008; Konstantinopoulos et al., 2010; Reis-Filho and Pusztai, 2011). In these studies, subtypes of breast and glioblastoma cancers have been identified. Another popular type of data that is used for tumor stratification is somatic mutation profiles. Similarities and differences in patient tumor mutation profiles provide information for tumor subtype stratification. However, some challenges have emerged since the somatic mutation profiles are sparse and heterogeneous. To overcome these challenges, some studies used network-based approach to discover cancer subtypes (Hofree et al., 2013; Hou and Ma, 2014; Jin et al., 2015; Kim et al., 2016; Cho et al., 2016; Zhang et al., 2018; Hu et al., 2020).

Hofree et al. introduced an approach named network-based stratification (NBS) to stratify tumors into meaningful subtypes by clustering patients who have mutations in similar network regions (Hofree et al., 2013). NBS is known as the first method in which somatic mutation profiles have been used for stratifying patients. This method integrates gene networks with tumor molecular profiles, and it considers the sparsity of mutations at network level. NBS is used to identify subgroups of patients by spreading the influence of each mutation profile in a gene interaction network. The main algorithm of NBS is a network propagation (Cowen et al., 2017) that uses a random walk model (Random Walk with Restart, RWR) (Pearson et al., 1905). In Hofree et al.’s study, to derive a stratification of the input cohort, a variant of NMF (GNMF) (Cai et al., 2011) was used; to identify robust cluster assignments, the technique of consensus clustering (Monti et al., 2003) was used. In 2018, the NBS algorithm was implemented as a python package. This package, called pyNBS, modularized the main steps of the algorithm and allowed better control and easier analysis (Huang et al., 2018).

Zhang et al. introduced a supervised method named network-based supervised stratification (NBS^2^) to classify tumors (Zhang et al., 2018). It extends the supervised random walk algorithm (Backstrom and Leskovec, 2011) by including a new loss function used for classification of cancer subtypes. NBS^2^ uses the network propagation technique for aggregation of mutations affecting the same subnetwork regions, same as other methods. Unlike these methods, NBS^2^ uses supervised learning to adjust the weight of each molecular interaction.

Besides, there are some studies that use a network embedding based stratification methodology to identify patient subtypes from somatic mutation profiles (Xu, et al., 2020; Rohani and Eslahchi, 2020).

Although all these existing studies provide valuable contributions to cancer subtype discovery, they have some limitations. In studies that stratify tumors into subtypes using somatic mutation profiles, the use of RWR method may cause an ill-conditioning problem. In general, “ill-conditioned problem” refers to the problem of inverting a matrix, which may have zero or close to zero rows or columns. Since the random walk procedure used in NBS and NBS^2^ (Hofree et al., 2013; Zhang et al., 2018) is a matrix inversion operation (Coskun et al., 2018), small degree nodes in the gene-gene network may cause this ill-conditioning problem. These small degree nodes in the random walk procedure also cause the sparsity problem as a result of the above-mentioned ill-conditioned problem. In this study, our aim is to elude this ill-conditioning problem with our proposed approach. Deep learning network embedding-based methods have classified tumor subtypes using supervised learning; however, these embedding methods entail heavy computational costs associated with training of neural networks or high order proximity measure computation (Coskun and Koyuturk, 2021). Compared to the existing approaches, our method offers an effective and efficient solution for tumor stratification problem.

## 2. Methods

In precision oncology, one of the crucial steps is biomolecule-based cancer subtype discovery, which can be basically divided into two categories as supervised and unsupervised methods. Supervised methods classify tumors into predefined subtypes using labeled molecular data sets, whereas unsupervised methods cluster patients (tumors) into classes or subcategories. Obtaining labeled data is mostly difficult, costly, and time consuming. On the other hand, unlabeled molecular data are easy to access; and thus, unsupervised methods have been employed for the tumor stratification problem. While solely using molecular data has been shown to be effective for tumor stratification, heterogeneity of this data limits the ability of the clustering methods (Alashwal et al., 2019). Thus, additional protein-protein interaction network information has been invoked to smooth (via gaining strength from known protein-protein interactions) and resolve the heterogeneity problem of the data.

In this paper, we propose an unsupervised learning method for stratification of cancer into meaningful subtypes, and it integrates somatic mutation profiles with the knowledge of the molecular interaction network. In the subsequent subsections, we first summarize the existing approaches for the unsupervised tumor stratification problem. We then introduce our method’s mathematical background.

### 2.1. Existing approaches

In the context of biology, various machine learning techniques have been used for different purposes such as diagnosis, prognosis, screening, or treatment in cancer (Kourou et al., 2014). Among these computational techniques, stratification and supervised classification are well-adopted for molecule-based cancer subtype discovery, which is an important field in precision medicine. In a general setting, somatic mutation profiles are combined with molecular network information in network-based tumor stratification supervised or unsupervised approaches. Using the network propagation techniques, such as RWR, the influence of each somatic mutation profile is propagated across its network neighborhood, termed as “smoothing”; and then clustering approaches are applied over the smoothed somatic mutation profiles. The clustering procedure operates as follows: Firstly, a certain part of the rows (patients or tumors) and columns (mutated genes) of the binary somatic mutation data are subsampled at random without replacement. Secondly, binary somatic mutation data is propagated over the network. Thirdly, the quantile normalization technique is applied to this network smoothed mutation data. Fourthly, graph (or network) regularized non-negative matrix factorization (GNMF) is used to decompose network data into k clusters. Finally, consensus clustering is applied over network smoothed mutation profiles.

#### 2.1.1. Network smoothing

In terms of tumor stratification, network propagation has been used for serving the same purpose, i.e., to capture the similarity among the nodes in the molecular network and to smooth the mutation signal across the network (Vanunu et al., 2010; Hofree et al., 2013). The basic idea behind network propagation is to employ a random walk (Pearson et al., 1905) model to diffuse information about tumor mutations using molecular interaction networks associations. Mathematically, we can define the network propagation as follows:


(1) $${\text{F}}_{\text{t}+1}=\alpha {\text{F}}_{\text{t}}\text{A}+(1-\alpha ){\text{F}}_{0}$$

F_0_ (patient-by-gene binary matrix) represents the mutation profile of each tumor. A is a degree normalized adjacency matrix of the molecular interaction network. The parameter α controls the random walker that determines how much a mutation signal should diffuse on the network. It is set in the range of 0 and 1. Iterative computation is performed via t values (0, 1, 2, …) until F_t+1_ converges. At convergence (F_t+1_ ≈ F_t_), F_t_ (propagated mutation profiles) denotes a patient -by- gene binary matrix in which each tumor’s mutation profile has been smoothed across the network. After obtaining the propagated mutation profiles, which might be high dimensional, non-negative matrix factorization is applied to this matrix to reduce its dimensionality and identify clinically and biologically meaningful subtypes (Hofree et al., 2013; Zhang et al., 2018).

#### 2.1.2. Nonnegative matrix factorization (NMF)

Nonnegative matrix factorization (NMF) is one of the dimensionality reduction techniques and it has been used for many learning tasks in which the following constraint holds, i.e., lower dimensional matrices must be positive (Cai et al., 2011). In the context of unsupervised tumor stratification problem, graph (or network) regularized version of non-negative matrix factorization (GNMF) is used to minimize the objective function given in the Equation 2 (Cai et al., 2008; Hofree et al., 2013; Huang et al., 2018).


(2) $${\left\|\text{F - WH}\right\|}^{2}+\lambda \text{Tr}({\text{HLH}}^{\text{T}})$$

In this equation, W and H are a decomposition of F (patient-by-gene matrix), which are formed as a result of network smoothing. W (genes-by-k) is the basis matrix and H (k-by-patients) is the patient cluster matrix. Tr() represents the trace of a matrix L = D − A is the graph Laplacian of the K-nearest-neighbor network, where D denotes diagonal degree matrix of the KNN network, and A is the adjacency matrix of the KNN network. λ is the regularization constant to scale network regularizer (L) term in GNMF (Cai et al., 2008; Cai et al., 2011). Multiple instances of H will be combined together during the consensus clustering step of the algorithm (Huang et al., 2018).

#### 2.1.3. Consensus clustering

This methodology serves to represent a consensus among multiple runs of the clustering algorithm. Also, it is used to detect the number of clusters in the data and evaluates the stability of the identified clusters. To represent the agreement among the clustering, a consensus matrix (N × N) is defined, where N denotes the number of elements in a dataset. A consensus matrix is calculated for each cluster, and each element in the matrix denotes the proportion of clustering runs in which two samples are clustered together (Monti et al., 2003). In the unsupervised tumor stratification problem, consensus clustering is achieved to produce final subtypes. GNMF is performed multiple times on subsamples of the dataset. Then, the set of clustering outcomes is transformed into a coclustering matrix. Hence, a patient linkage map is created from this coclustering matrix and patient clusters are assigned using the patient link map hierarchy.

On the other hand, supervised methods classify tumors into predefined subtypes using labeled datasets. Inspired by the supervised random walk approach presented in (Backstrom and Leskovec, 2011), cancer classification is used to identify potential biomarkers as well as to predict patient survival times and cancer prognosis. To this end, NBS^2^ learns the center of each subtype by using subtype information as labels and similarly labeled smoothed profiles are forced to stay in same clusters (Zhang et al., 2018).

### 2.2. Our approach

**Figure 1:**
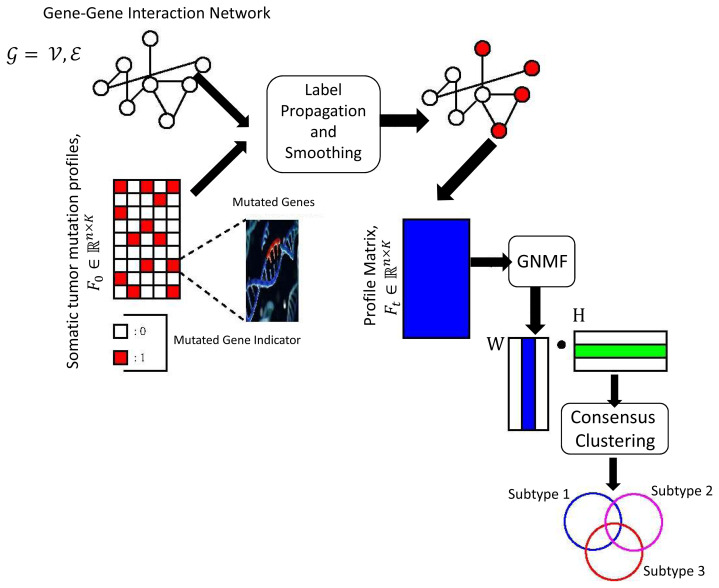
Overview of the proposed label propagation-based tumor stratification algorithm. 
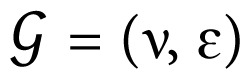
 denotes gene-gene interaction network, F_0_ shows somatic tumor mutation profiles. We first apply label propagation to the network by (L + σI)^−1^ then smooth the profiles with this inverted matrix, by just matrix-vector multiplication. We then apply graph regularized nonnegative matrix factorization to smoothed profile to eliminate linearly depended columns of it. Finally, we use consensus clustering to obtain subtypes.

As illustrated in Figure 1, the basic premise behind our proposed approach can be summarized in simple terms as follows. We start with the somatic mutation data, which has rows consisting of 1 and 0 corresponding to the mutated genes and nonmutated genes respectively. We use these mutated genes within the label propagation algorithm that we proposed in this study. In this way, we calculate the topological similarities of the genes in the gene-gene network to determine the most relevant genes as compared to the mutated genes. Subsequently, we construct a gene profile matrix F_t_ based on the label propagation algorithm and this profile matrix encodes the relevance scores of the mutated genes. However, this profile matrix can be high dimensional, in other words, there could be linearly dependent columns. To alleviate this problem, we use nonnegative-matrix factorization to obtain an intrinsic representation of the profile matrix and we finally cluster the subtypes based on this final intrinsic matrix.

Our proposed method could be presented mathematically as following. By considering F_0_ Є R^nxK^ (gene-by-tumor binary matrix) as a label set matrix, where K << n we can define the tumor stratification problem as a well-known label propagation algorithm (Zhu and Ghahramani, 2002). To be more specific, we will use the indicator values of each column of the F_0_ matrix as a label of that tumor. To this end, we will define known labels as Y_L_ = (y_1_, … … y_K_), and unknown labels as Y_U_ = (y_K+1_, … … y_K+U=n_). Now, our objective is to determine the set of Y_U_ by depending on Y_L_ and the graph’s topological structure. To be more consistent with the terminology, we will call Y_L_ = F_0_.

By using the above defined F_0_ Є R^nxK^ (gene-by-gene binary matrix) as known labels in our setting, we will define the tumor stratification problem as a label propagation approach. Let F_0_ Є R^nxK^ denote a label information matrix, where 1 shows the cluster index. In this study, we assume the manifold smoothness of known labels, i.e, the similar nodes in graph should share similar labels; and penalize the sparseness of unknown labels $\left(\hat{{\text{f}}_{0}}\right)$, i.e., low degree nodes should be penalized. In this context, smoothness simply refers to the multiplication of the adjacency matrix (column normalized adjacency matrix) of the gene-gene integration network with the somatic mutation data. Hence, smoothness operation helps us to consider somatic mutation and gene-gene interactions collectively and simultaneously. In this way, we take into account not only the labeled nodes, but also their neighbors in the gene-gene interaction network. Inspired by the assortativity term in our previous study (Coskun et al., 2019), mathematically we can define the smoothness as follows:


(3) $$\begin{array}{l} \text{Smoothness }\left(\hat{{\text{f}}_{0}}\right)=\sum_{\text{i},\text{j}=1}^{\text{n}}{\text{A}}_{\text{i},\text{j}}\;{\left({\text{f}}_{0_{\text{i}}}-{\hat{{\text{f}}_{0}}}_{\text{j}}\right)}^{2}\\ =\Sigma_{\text{i},\text{j}=1}^{\text{n}}{\text{A}}_{\text{i},\text{j}}\;({{\text{f}}_{0_{\text{i}}}}^{2}-2{\text{f}}_{0_{\text{i}}}{\hat{{\text{f}}_{0}}}_{\text{j}}+{{\hat{{\text{f}}_{0}}}_{\text{j}}}^{2})\\ =2{{\hat{{\text{f}}_{0}}}_{\text{j}}}^{\text{T}}(\text{D-A}){\text{f}}_{0_{\text{i}}}\\ =2\hat{{\text{f}}_{0}}{\text{Lf}}_{0} \end{array}$$

Here, L denotes the graph Laplacian, and it is defined as L = D − A. A is the degree normalized adjacency matrix of the interaction network and D represents the diagonal degree matrix of A.

The sparsity of the assignments can be measured as follows:

Formalization of the objective function is obtained by combining these two equations. Hence, we have the following objective function:


(4) $$\begin{array}{l} \text{Sparsity }\left(\hat{{\text{f}}_{0}}\right)=\sum_{\text{i}=1}^{\text{n}}{\left({\hat{{\text{f}}_{0}}}_{\text{i}}\right)}^{2}\\ ={||\hat{{\text{f}}_{0}}||}^{2} \end{array}$$

(5) $$\text{J}(\hat{{\text{f}}_{0}})=\hat{{\text{f}}_{0}}{\text{Lf}}_{0}+\sigma {\left\|\hat{{\text{f}}_{0}}\right\|}^{2}$$

Here, we aim to find the $\hat{{\text{f}}_{0}}$ that minimizes $\text{J}\left(\hat{{\text{f}}_{0}}\right)$. The final renders us eliminating zero rows/columns of the matrix we are inverting. The parameter σ configures the effect of this penalization. By taking the partial derivative of J with re spect to $\hat{{\text{f}}_{0}}$, the following equation is obtained:


(6) $$\frac{\partial J}{\partial \hat{{\text{f}}_{0}}}=\hat{{\text{f}}_{0}}\text{L}+\sigma \hat{{\text{f}}_{0}}$$

If the Equation 6 is set to 0, then $\hat{{\text{f}}_{0}}$ that minimizes can $\text{J}\left(\hat{{\text{f}}_{0}}\right)$ be calculated as:


(7) $$\hat{{\text{f}}_{0}}={\left(\text{L}+\sigma \text{I}\right)}^{-1}{\text{f}}_{0}$$

Now, by using the above equation, we try to find the clusters of $\hat{{\text{f}}_{0}}$ by relying on the Laplacian graph, and we stratify the tumors. Our approach here is a reflection of the Ridge regularization on tumor clustering:

Firstly, inspired by our previous study (Coskun et al., 2018) we rewrite the RWR equation as follows:


(8) $${\text{F}}_{\text{t}+1}=\alpha {\left(\text{I}-(1-\alpha )\text{A}\right)}^{-1}{\text{F}}_{0}$$

Hofree et al. use random walk model to diffuse information about tumor mutations using molecular interaction knowledge in the network. Instead, in this study, we propose the label propagation approach (as presented above) by replacing α(I-(1−α)A) with L + σI, so that we can elude the above-mentioned ill-conditioned problem. In the random walk procedure, the small degree nodes in the gene-gene network also cause the sparsity problem as a result of the ill-conditioned problem. To circumvent this ill-conditioning problem, we shift the Laplacian matrix via σI, where I is an identity matrix. Finally, we define the diffuse strategy of tumor mutations using the knowledge of the molecular interactions as follows:


(9) $${\text{F}}_{\text{t}+1}={\left(\text{L}+\sigma \text{I}\right)}^{-1}{\text{F}}_{0}$$

In the Equation 9, the parameter σ is set to 0.01, 0.1 and 0.2. After obtaining the smoothed mutation profiles, we apply non-negative matrix factorization to eliminate the high dimensionality problem in the smoothed somatic profiles. Finally, clustering is performed based on this intrinsic representation of the smoothed profiles. The steps of our proposed approach are illustrated in Figure 1.

### 2.3. Clinical analysis

In order to evaluate the relationship between the identified clusters and the clinical data, we have performed survival analysis using the “lifelines” package in Python. In order to investigate the association between the subtypes and patient 5-year survival time, Kaplan–Meier survival curves of subtypes were generated and log-rank test p-values were calculated.

## 3. Results

To evaluate the performance of our method, the results are compared against the state-of-the-art unsupervised and supervised methods. Our method was tested on colon (colorectal adenocarcinoma), uterine (uterine corpus endometrial carcinoma), head and neck (head-neck squamous cell carcinoma), and bladder (urothelial bladder carcinoma) datasets obtained from TCGA. The comparative evaluation of our method with the NBS method (unsupervised) is presented in Section 3.1. Our method was also tested on the breast cancer dataset, obtained from TCGA; and the comparative evaluation of our method with the validation performance of NBS^2^ method (supervised) are also presented in Section 3.1. The clinical analysis that we conducted to evaluate whether our method and state-of-the-art methods can stratify cancer types into clinically informative subtypes is given in Section 3.2. We followed the same subsampling procedure presented in NBS (Hoffree et al., 2013), where the subsampling implies the removal of the genes that contain less than ten mutations. Similarly, in our experiments, 80% of the somatic mutation matrix rows and columns were subsampled without replacement. Graph regularized NMF (GNMF) was performed 100 times on the subsamples of the dataset to stratify the input cohort. To generate robust patient clusters, consensus clustering is used, and hence, a final stratification of the patients into clusters is obtained. Aggregate GNMF results of 100 samples were converted into a co-clustering matrix. Each element in this matrix represents the frequency of that tumor pair as assigned to the same cluster, among all clustering iterations.

### 3.1. Benchmarking of clustering performance with state-of-the-art methods

In this paper, we have compared our approach (unsupervised label propagation) with existing supervised and unsupervised approaches.

Unsupervised approach: To make a fair comparison with NBS (Hofree et al., 2013), we evaluate our proposed method using the same datasets that are utilized in Huang et al., 2018, the manuscript on the python implementation of NBS algorithm. We applied both our method and the NBS method to cluster somatic mutation profiles of four different cancer types: colon (COAD), uterine (UCEC), head and neck (HNSC), and bladder (BLCA). The numbers of the tumors and the numbers of the genes for each one of the tumor mutation datasets are shown in Table 1. As a reference molecular network, we used a filtered network, which has to preserve only cancer genes. This filtered network has 2291 nodes (Huang et al., 2018).

To evaluate the clustering performances, we used Silhouette Coefficient (Rousseeuw, 1987), Davies–Bouldin index (Davies and Bouldin, 1979), and intra-cluster distance (Bolshakova and Azuaje, 2003).

In this study, each method was run five times. For each run, the Silhouette coefficient and Davies–Bouldin index values were calculated, and the results were displayed using the boxplots. Higher average Silhouette coefficient values and lower Davies–Bouldin index values indicate better clustering quality. Intra cluster distance values (complete, average, and centroid) are calculated separately for each extricated cluster. For a better assessment, the calculated values for each cluster were averaged and the results are shown by tables. Tables should be interpreted by keeping in mind that intra-cluster distance should be minimum to obtain the best clustering result. Therefore, the lowest distance in each column is highlighted in bold.

In order to visualize the clusters, we used t-SNE (t-distributed stochastic neighbor embedding) (Mateen, 2008) and heatmap of the co-clustering matrix (co-clustering map) (Monti et al., 2003). t-SNE is a nonlinear dimension reduction technique, and it is a commonly used graphic approach to assist clustering methods such as k-means, with respect to determining the number of clusters and cluster memberships. In our study, t-SNE method is employed for visualizing the identified clusters (subtypes of tumors); and comparing those identified clusters among different methods. To this end, based on the somatic mutation profiles of four different cancer types, two-dimensional t-SNE maps were generated separately for our method using three different σ parameters and for NBS method. Using the clustering results of each method, we have also generated heatmaps of the co-clustering matrix (co-clustering maps) for each of the four datasets separately.

#### 3.1.1. Clustering of colon cancer (COAD)

**Figure 2:**
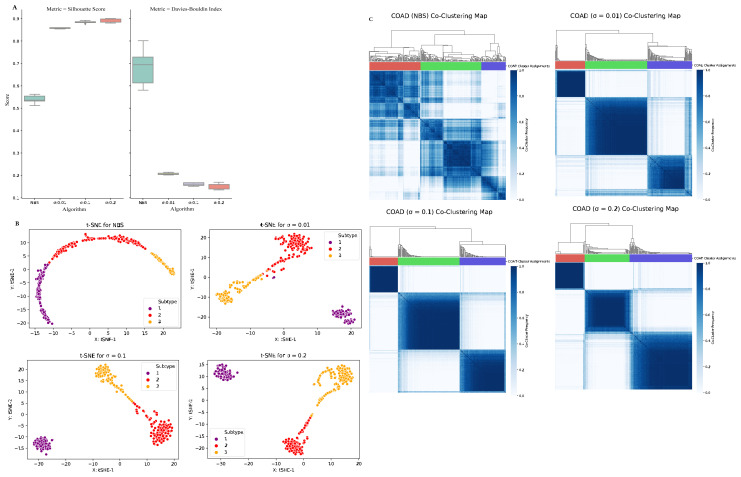
Performance evaluation of the methods (NBS, our proposed method with three different σ parameters) on the COAD dataset. (A) Comparative evaluation of the methods using Silhouette coefficient and Davies–Bouldin index scores, which are obtained by running each method five times. (B) Two-dimensional t-SNE maps of the identified clusters by different methods. (C) Heatmaps of the coclustering matrix (coclustering maps) generated using the clustering results of each method.

By applying the NBS and our method to colon cancer data, patient profiles are clustered into three predefined subtypes. The calculated intra cluster distance values (complete, average, and centroid) of COAD dataset are presented in Table 2 for the NBS method, and for our method (with three different σ parameters). Figure 2 shows the performances of tested clustering methods on colon cancer (COAD) dataset. The boxplot in Figure 2A displays the comparative evaluation of the Silhouette coefficient and Davies–Bouldin index scores, that are obtained by running each method five times. On the COAD dataset, for all three different σ parameters, our method yielded higher Silhouette coefficient values and lower Davies–Bouldin index scores compared with the NBS method (Figure 2A). This finding implies that on the COAD dataset, our method performs better cluster identification compared to the NBS method. The t-SNE plots in Figure 2B indicate that when the tumor mutation profiles of COAD patients are stratified using our method, three clusters are well grouped among themselves. The co-clustering maps in Figure 2C visualizes three clearly separated blue blocks along the diagonal for our method for all σ values. These maps demonstrate well-defined three-cluster structure. On the other hand, for NBS, there are more than three blue blocks, and the blocks are not visually separable.

#### 3.1.2. Clustering of uterine cancer (UCEC)

**Figure 3:**
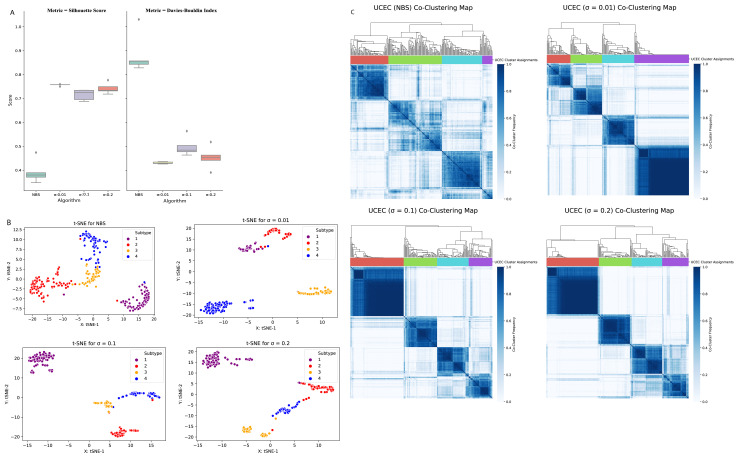
Performance evaluation of the methods (NBS, our proposed method with three different σ parameters) on the UCEC dataset. (A) Comparative evaluation of the methods using Silhouette coefficient and Davies–Bouldin index scores, which are obtained by running each method five times. (B) Two-dimensional t-SNE maps of the identified clusters by different methods. (C) Heatmaps of the coclustering matrix (coclustering maps) generated using the clustering results of each method.

Using uterine cancer data, patient profiles are clustered into four predefined subtypes. The calculated intra cluster distance values (complete, average, and centroid) of UCEC dataset are presented in Table 3 for the NBS method and for our method (with three different σ parameters). Figure 3 displays the performances of tested clustering methods on the UCEC dataset. The boxplot in Figure 3A comparatively evaluates the Silhouette coefficient and Davies–Bouldin index scores, which are obtained by running each method five times. On the UCEC dataset, for all three different σ parameters, our method resulted in higher Silhouette coefficient values and lower Davies–Bouldin index scores compared to the the ones obtained by NBS method (Figure 3A). This finding implies that on the UCEC dataset, our method performs better cluster identification compared to the NBS method. The t-SNE plots in Figure 3B imply that when the tumor mutation profiles of UCEC patients are stratified using our method, four clusters are well grouped among themselves. The coclustering maps in Figure 3C visualize four clearly separated blue blocks along the diagonal for our method for all σ values. These maps demonstrate well-defined four-cluster structure. On the other hand, the blocks are not visually separable in the co-clustering map of the NBS method for UCEC data.

#### 3.1.3. Clustering of head and neck cancer (HNSC)

**Figure 4:**
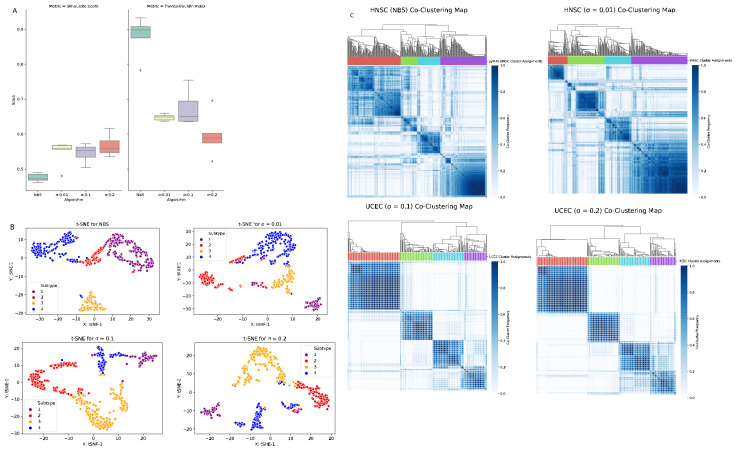
Performance evaluation of the methods (NBS, our proposed method with three different σ parameters) on the HNSC dataset. (A) Comparative evaluation of the methods using Silhouette coefficient and Davies–Bouldin index scores, which are obtained by running each method five times. (B) Two-dimensional t-SNE maps of the identified clusters by different methods. (C) Heatmaps of the coclustering matrix (coclustering maps) generated using the clustering results of each method.

Using head and neck cancer data, patient profiles are clustered into four predefined subtypes. The calculated intra cluster distance values (complete, average, and centroid) of HNSC dataset are presented in Table 4 for the NBS method, and for our method (with three different σ parameters). Figure 4 displays the performances of tested clustering methods on the HNSC dataset. The boxplot in Figure 4A comparatively evaluates the Silhouette coefficient and Davies-Bouldin index scores, which are obtained by running each method five times. On the HNSC dataset, for all three different σ parameters, our method yielded higher Silhouette coefficient values and lower Davies–Bouldin index scores compared with the NBS method. This finding implies that, on the HNSC dataset, our method performs better cluster identification compared to the NBS method. The t-SNE plots in Figure 4B imply that when the tumor mutation profiles of HNSC patients are stratified using our method, four clusters are well grouped among themselves. For this dataset, although there is no clear difference between the co-clustering maps presented in Figure 4C, in our method, the four blue blocks are more distinguishable compared to the NBS method.

#### 3.1.4. Clustering of bladder cancer (BLCA)

**Figure 5:**
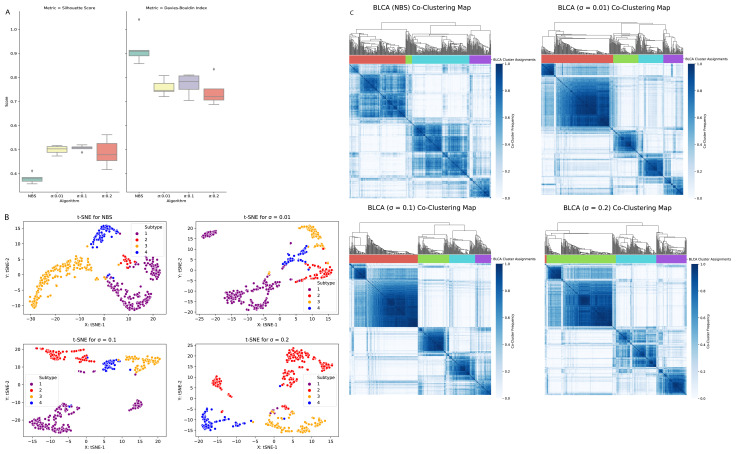
Performance evaluation of the methods (NBS, our proposed method with three different σ parameters) on the BLCA dataset. (A) Comparative evaluation of the methods using Silhouette coefficient and Davies–Bouldin index scores, which are obtained by running each method five times. (B) Two-dimensional t-SNE maps of the identified clusters by different methods. (C) Heatmaps of the coclustering matrix (coclustering maps) generated using the clustering results of each method.

When the NBS method and our method are applied to the bladder cancer data, patient profiles are clustered into four predefined subtypes. The calculated intra cluster distance values (complete, average, and centroid) of BLCA dataset are presented in Table 5 for the NBS method, and for our method (with three different σ parameters). Figure 5 illustrates the performances of tested clustering methods on the BLCA dataset. The boxplot in Figure 5A displays the comparative evaluation of the Silhouette coefficient and Davies–Bouldin index scores, which are obtained by running each method five times. On the BLCA dataset, for all three different σ parameters, our method yielded higher Silhouette coefficient values and lower Davies-Bouldin index scores compared with the NBS method. This finding implies that on the BLCA dataset, our method performs better cluster identification compared to the NBS method. The t-SNE plots in Figure 5B imply that when the tumor mutation profiles of BLCA patients are stratified using our method, four clusters are well grouped among themselves. For this dataset, although there is no clear difference between the co-clustering maps presented in Figure 5C, in our method, the four blue blocks are more distinguishable compared to the NBS method.

**Figure 6:**
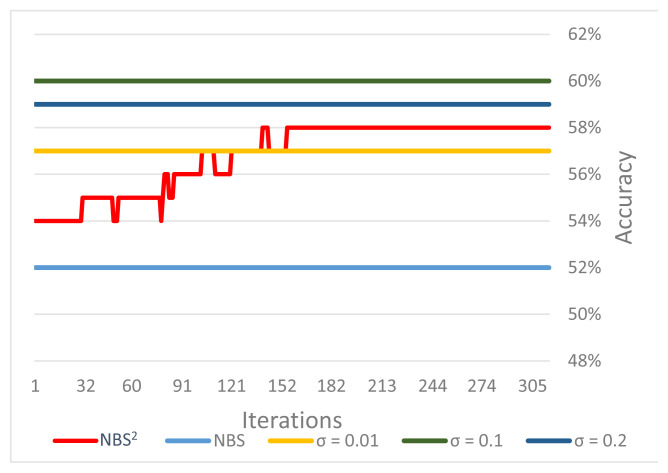
Performances of algorithms for breast cancer subtype identification.

Supervised approach: In this section, using breast cancer dataset, we have assessed the performance of our proposed method against the performance of network-based supervised stratification (NBS^2^), which is the baseline supervised method for cancer subtype identification (Zhang et al., 2018). We compared the validation performance of NBS^2^ with the performance of our method. While the training dataset used for the NBS^2^ method contains 577 tumors and 571 genes, the validation dataset contains 286 tumors and 571 genes. The reference molecular network containing 557 genes was used. At the end of 316 iterations, the accuracy of the tumor validation set is increased from 54% to 58%. It should be noted that it is not easy to compare a supervised method with an unsupervised method comprehensively. Hence, in order to compare our unsupervised method with the NBS^2^, we followed the assessment technique proposed in (Zhang et al., 2018). To make validation set predictions, NBS^2^ calculates the cluster centroids of the training set. In order to evaluate the performances of algorithms, on the validation data, we have plotted the classification accuracy against the number of NBS2 iterations. As shown in Figure 6, our method achieved 60% accuracy as the best score, which is 2% higher than that of NBS^2^. As expected, the accuracy values of the unsupervised methods are stable over iterations. We can imply from Figure 6 that on the breast cancer dataset, in terms of accuracy, the performance of our proposed unsupervised learning algorithm is better than both the NBS method (unsupervised algorithm) and NBS^2^ method (supervised algorithm).

### 3.2. Clinical analysis

**Figure 7:**
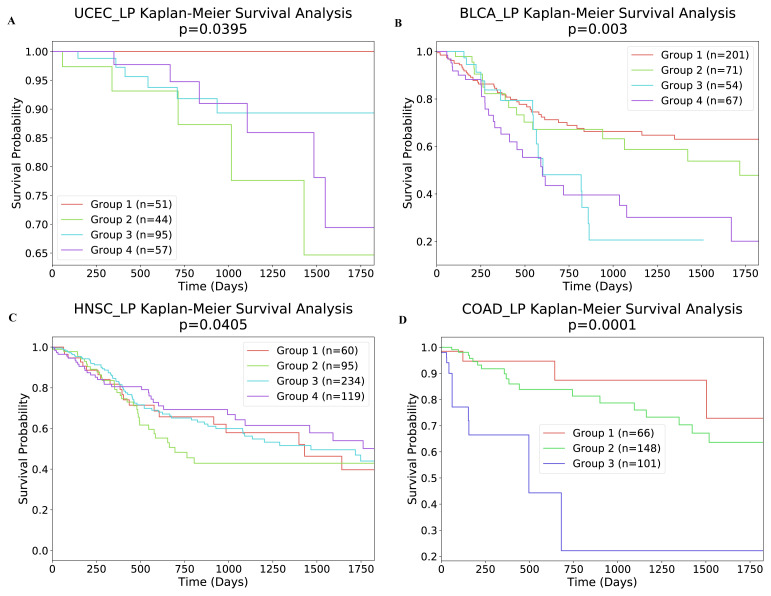
Survival analysis of four cancers with our label propagation-based method with σ = 0.01. (A) Kaplan–Meier survival plots for UCEC. (B) Kaplan–Meier survival plots for BLCA. (C) Kaplan–Meier survival plots for HNSC. (D) Kaplan–Meier survival plots for COAD.

**Figure 8:**
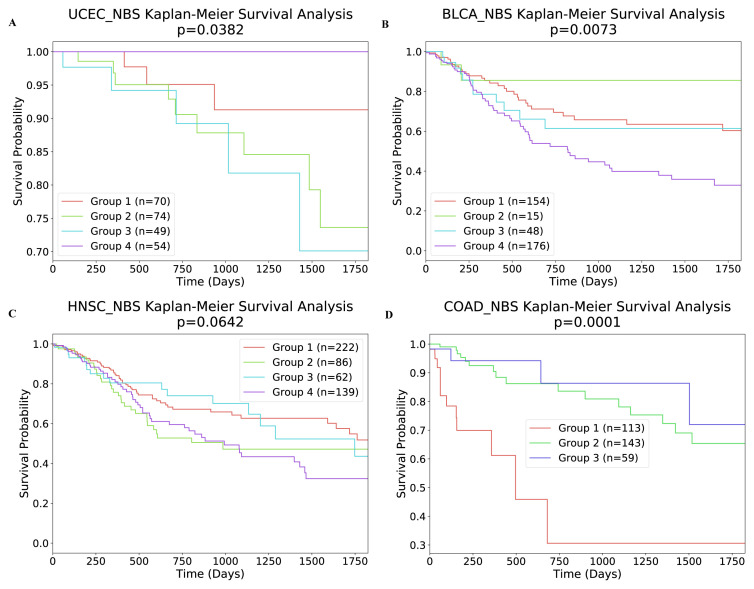
Survival analysis of four cancers with unsupervised NBS method (Hofree et al., 2013). (A) Kaplan–Meier survival plots for UCEC. (B) Kaplan–Meier survival plots for BLCA. (C) Kaplan–Meier survival plots for HNSC. (D) Kaplan–Meier survival plots for COAD.

**Figure 9:**
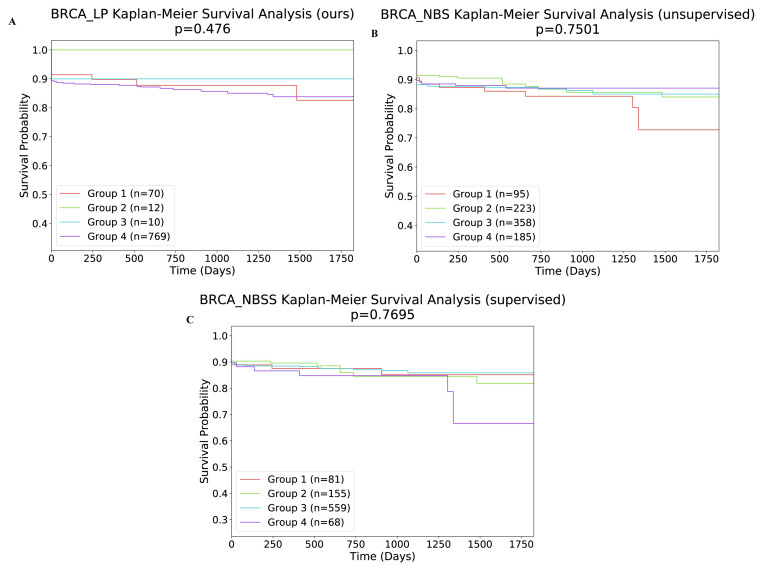
Survival analysis of breast cancer with (A) our approach (unsupervised), (B) NBS method (unsupervised) (Hofree et al., 2013), and (C) NBS2 (supervised) (Zhang et al., 2018).

**Figure 10:**
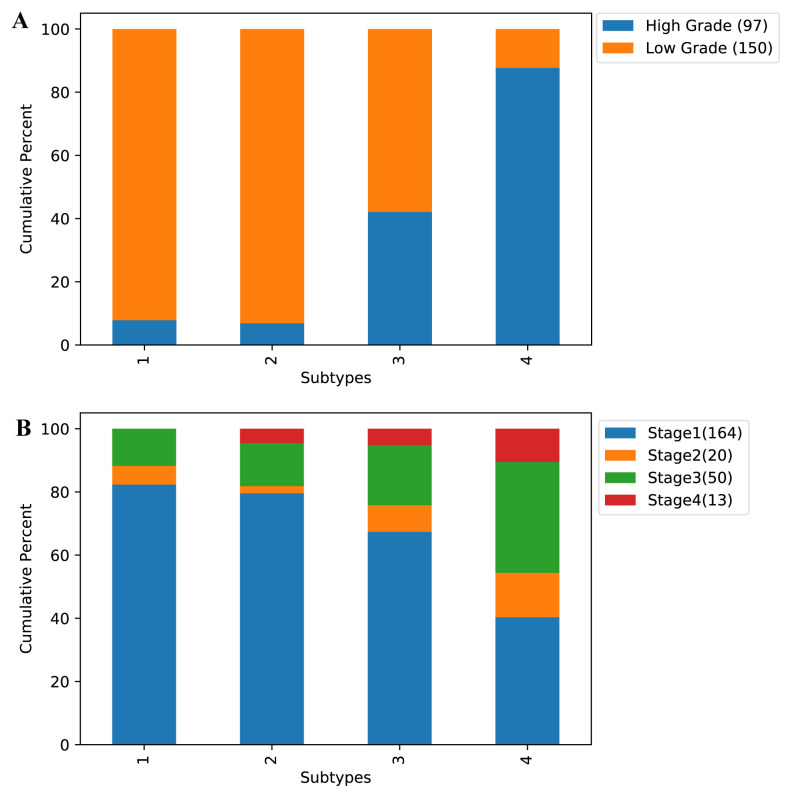
Composition of the identified subtypes in terms of (A) tumor grade and (B) tumor stage for UCEC. There are 51, 44, 95, 57 patients in Subtypes 1, 2, 3, 4, respectively.

In this section, to assess the biological significance of the identified clusters, we examined whether the identified subtypes were predictive of clinical data. Firstly, we investigated the relationship between the subtypes and 5-year survival. As shown in Table 6, all four cancer subtypes identified by our method were significantly associated with survival (at p < 0.05 level). In Figure 7, we present the Kaplan–Meier survival curves of the identified clusters for UCEC, BLCA, HNSC, COAD samples. We can conclude from Figure 7 that each cluster was independent and differed in survival. We then perform the same experiment with an unsupervised approach, NBS (Hofree et al., 2013). The result of this analysis is shown in Figure 8. As it can be seen in the figure, unsupervised method delivers higher p-value comparing to that of our approach except UCEC. Furthermore, we assess the clinical survival rate performance of supervised and unsupervised methods on breast cancer data. The resulting analysis is depicted in Figure 9. As shown in the figure, our method (unsupervised) outperforms both supervised and unsupervised existing methods (Zhang et al., 2018; Hofree et al., 2013) with lower p-value. We note that we are only able to perform supervised analysis on this breast cancer data since it is the only available labelled data. Next, we analyzed the clinical characteristics of the four identified subtypes of UCEC. In Figure 10, we present the composition of the identified subtypes in terms of tumor grade and tumor stage for UCEC. One can observe from Figure 10A that while the identified Subtype 1 and 2 is mainly populated with low grade-patients, the patients in Subtype 4 are mainly high grade. As shown in Figure 10B, there are no Stage 4 patients in the identified Subtype 1, and this group of patients have better survival as shown in Figure 7A with brown color. On the other hand, we can imply from Figure 10B that Subtype 2, 3, and 4 groups include higher numbers of Stage 3 and 4 patients; the patients in these subtypes have lower survival probability (as shown in green, blue, and purple in Figure 7A). Based on these analyses, we can suggest that better computational separation could be associated with more meaningful clinical groups.

## 4. Discussion

In this study, we present a new label propagation based unsupervised method to stratify tumor mutation profiles into meaningful subtypes. In order to evaluate the performance of the proposed method, we have conducted experiments on five different cancer types. The proposed method is comparatively evaluated against the network-based stratification method (NBS) (Hofree et al., 2013), which is an unsupervised method. According to the performance evaluation results presented in Section 3.1, our label propagation-based method (for all three σ values) drastically outperforms the NBS method in subtype stratification. We have observed that the σ parameter can be tuned. Additionally, we have performed a comparison with a supervised method (NBS^2^, proposed by **(**Zhang et al., 2018) using breast cancer dataset. The results presented in second part of Section 3.1 (supervised approach) imply that we obtained comparable performance metrics. Taking into account that our method is an unsupervised method, here we report a promising result where the performance of subtype identification without using any label is comparable with the performance of subtype classification using labels. Supervised methods use labelled datasets and node-labelling is often expensive and time consuming. Hence, a method that can show a competitive performance even without using any label would be very useful for the identification of cancer subtypes.

We would like to note that different mutations on the same gene might have different effects. In literature (Hofree et al., 2013), it has been reported that the similarities and differences in patient tumor mutation profiles provide valuable information, and they have been studied for tumor subtype stratification. While some of the studies make use of somatic mutation profiles at the gene level, some others take into account the effect of different mutations on the same gene. In this study, we focused on the development of an alternative unsupervised method (a label propagation-based approach) to stratify cancer patients into subtypes using applied numerical algebra techniques and somatic mutation profiles at the gene level. To make a fair comparison, we have evaluated the performance of our method against NBS and NBS^2^, both of which use somatic mutation profiles at the gene level, using the same four datasets that are utilized in (Huang et al., 2018). We plan to incorporate mutation level data into our proposed method as a future work.

As another future study, we plan to extend the idea presented in this paper to the node-embedding context, where we have listed the related works as (Xu, et al., 2020; Rohani and Eslahchi, 2020). Since our proposed method in this article is a direct application of the random walk, to make a fair comparison, here we compare our method against other random walk-based approaches such as NBS and NBS^2^. In the future, we would like to work on the effects of node embedding, such as SiGraC, (Coskun and Koyuturk 2021) on subtype clustering problems. Thus, we left the node embedding based comparisons with (Xu, et al., 2020; Rohani and Eslahchi, 2020) as a future work.

## 5. Conclusion

In cancer, response to treatment varies from patient to patient. This variability emerges from the characteristics of patient tumor mutation profiles, and hence there is an utmost need to cluster patients based on their genomic similarities. Discovering cancer subtypes is one of the active research topics in cancer informatics. To date, various approaches have been developed for cancer subtype identification using many types of omics data. In this paper, a new label-propagation based unsupervised method is presented to stratify tumor mutation profiles into meaningful subtypes. Using various datasets and extensive experimental configurations on these datasets, we show that our proposed approach outperforms the alternative methods of cancer subtype identification by a large margin.

## Figures and Tables

**Table 1:** Descriptive statistics of the datasets.

Dataset	Number of tumors	Number of genes
COAD (Colon)	315	17390
UCEC (Uterine)	248	17341
HNSC (Head and Neck)	510	16521
BLCA (Bladder)	395	17201

**Table 2:** Intracluster distances for COAD.

Distance	Complete	Average	Centroid
Algorithm			
NBS	7.906	3.038	2.274
σ = 0.01	6.180	1.193	1.110
σ = 0.1	**6.118**	**1.107**	**1.101**
σ = 0.2	7.372	1.516	1.213

**Table 3:** Intracluster distances for UCEC.

Distance	Complete	Average	Centroid
Algorithm			
NBS	5.581	2.658	1.870
σ = 0.01	**4.195**	**1.704**	**1.141**
σ = 0.1	4.385	1.841	1.256
σ = 0.2	4.853	1.963	1.384

**Table 4:** Intracluster distances for HNSC.

Distance	Complete	Average	Centroid
Algorithm			
NBS	8.967	3.732	2.738
σ = 0.01	8.202	**3.130**	**2.147**
σ = 0.1	**8.142**	3.207	2.214
σ = 0.2	8.396	3.389	2.309

**Table 5:** Intracluster distances for BLCA.

Distance	Complete	Average	Centroid
Algorithm			
NBS	6.624	2.371	1.677
σ = 0.01	**6.153**	2.290	**1.420**
σ = 0.1	6.182	**2.264**	1.494
σ = 0.2	6.480	2.300	1.501

**Table 6:** The relationship between subtypes and survival in four cancer types using NBS and our method.

		Survival p-value (log-rank test)	
Dataset	Cluster number	NBS	Our Approach (σ: 0.01)
BLCA	4	0.0073	0.0030
COAD	3	0.0001	0.0001
HNSC	4	0.0642	0.0405
UCEC	4	0.0382	0.0395

## Footnotes

1 NCI. Precision Medicine in Cancer Treatment [online]. Website: https://www.cancer.gov/about-cancer/treatment/types/precision-medicine [accessed September 17, 2020).
